# Permanganate of Potassium in Amenorrhœa

**Published:** 1885-04

**Authors:** Edmund J. Doering

**Affiliations:** 2406 Prairie Ave.; Chicago


THE CHICAGO

### Medical Journal and Examiner.

Vol. L. APRIL, 1885. No. 4.

### Original Communications.

### Article I.

Some Remarks on the Value of Permanganate of Potassium in Amenorrhœa. *By* Edmund J. Doering, M. D., *Chicago.*

[Inaugural Thesis, read before the Chicago Gynecological Society, March 20th, 1885.]

Although several years have elapsed since Drs. Ringer and Murrell first introduced permanganate of potash, as an emmenagogue, to the profession, there is yet a feeling of uncertainty as to its true value. I presume this is partly due to the difficulty of determining in any given case, whether the reappearance of the menses is due to the remedy given or due to natural causes; and on the other hand, one or two failures with a new remedy will generally cause the physician to abandon its further use. It is therefore only by carefully conducted clinical observations, and by the accumulated experience of those who have given this remedy a fair trial, that it can be finally determined whether permanganate of potash is or is not an efficient emmenagogue. According to the U. S. Pharmacopœia, permanganate of potash occurs in deep-purple, violet, or nearly black needle-shaped rhombic prisms of a metallic lustre, permanent in the air, odorless, having a sweet, afterward disagreeable, astringent taste, and a neutral reaction. It is soluble, with the exception of a scanty brown residue, in twenty parts of water at 59° Fahr. and in three parts of boiling water.

It is remarkable for the quantity of oxygen which it contains and for the readiness with which it yields it up, and upon this chemical fact its use is based, both as an internal and external remedy. On the other hand, this very fact is claimed by chemists as an absolute proof that it is utterly useless when administered by the stomach.

Bartholow, who has great faith in this drug, claims that, although it parts with great readiness with its oxygen, this readiness is *not* sufficiently great to prevent the distribution of this gas into the blood. His opponents deny this, and argue that the organic matter contained in the stomach and mucous membranes is sufficient to appropriate the oxygen of the salt and thus prevent its entrance into the circulation.

Desiring further light upon this matter, I requested Professor N. Gray Bartlett, the well-known chemist, to give his views, which he courteously consented to do. His opinion is as follows:

"From the readiness with which the permanganate of potssium is decomposed by organic compounds, it would seem to be ineligible for internal use. When so administered, it is immediately brought in contact, in the stomach, with a relatively large amount of organic matter, and must, necessarily, be very rapidly destroyed, the manganese of the permanganate separating, in all probability, in the form of the hydrated manganese dioxide. The latter compound is an active oxidizing agent, and is possibly capable of exercising in the economy the oxidizing function which has been ascribed to the permanganate of potassium. It would seem rational, therefore, anticipating the change which follows the administration of the permanganate, to substitute for the latter the hydrated manganese dioxide, which can readily be prepared in a state of purity for medicinal use."

Whatever view may be adopted of the chemical changes which the permanganate undergoes in the human economy, the main question is as to its therapeutical value. Professor Thomas, in a recent address to the New York State Medical Association, says: "Permanganate of potash, as an excitant of the menstrual flow, is, I think, the best emmenagogue which has yet been discovered. Drs. Ringer and Murrell recommend this remedy in amenorrhœa, depending on torpor, anæmia and deficient activity of the menstrual apparatus, it being contra-indicated when acute congestion or a general condition of sthenic reaction exists. They recommend it to be given in doses of one grain first, to be gradually increased to two grains four times a day, as near as possible to the regular date of menstruation, and continued for three or four days. I may say, right here, that in every case in which I have administered the drug in this manner, it has proven useless. I have given the permanganate of potash a careful trial, having used it in about thirty cases of amenorrhœa, depending on anæmia and general atony of the sexual apparatus. Of this number, I have eliminated about one-half, in the subjoined table, the action of the remedy not having been satisfactory, from various causes, such as inattention to the general directions, want of perseverance in taking the medicine, etc., so that the actual number of cases on which my conclusions are based, is narrowed down to fourteen, in each of which, however, the cause of the amenorrhœa was entirely clear, the remedy carefully and continuously given, and the effect closely observed. In order to be brief, I have grouped these cases in the following table, instead of giving the history of each case at length:

| CASE. | AGE. | Single or Married. | Cause of Amenorrhœa. | Length of time Catamenia abs. | Remedy, How Administered. | RESULTS. | REMARKS. |
|---|---|---|---|---|---|---|---|
| I. | 16 | S. | Anæmia. | 4 months. | 1 gr. 3 p. d. | Failure. | Remedy given for one week at date of expected menstruation for two consecutive months. Menses appeared finely under a course of iron tonics. |
| II. | 17 | S. | Anæmia. | 2 months. | 1 gr. 4 p. d. | Failure. | Similar result as above. |
| III. | 19 | S. | Anæmia. | 3 months. | " " | Failure. | " " |
| IV. | 26 | M. | General atony. | 3 months. | 2 gr. 3 p. d. | Success. | Remedy taken for three weeks. |
| V. | 20 | M. | Anæm. fr. nursing. | 4 months. | " " | Success. | " " one month. |
| VI. | 40 | S. | General atony. | 3 months. | 2 gr. 3 p. d. | Failure. | Remedy taken every alternate week for six weeks. Three weeks after discontinuing remedy, menses appeared. |
| VII | 17 | S. | " " | 2 months. | 1 gr. 3 p. d. | Failure. | Remedy given at expected period for two months. |
| VIII. | 18 | S. | Anæmia. | 2 months. | 2 gr. 3 p. d. | Success. | Remedy taken for two weeks. |
| IX. | 29 | M. | General atony. | 3 months. | 2 gr. 3 p. d. Later, 4 gr. 3 p. d. | Success. | Three weeks, two grains three times a day—no result. Finally, four grains three times a day, successful in one week. Next period again missed. At the expected period following, the drug given in four grain doses for one week, successful. Since then, period regular. |
| X. | 18 | S. | Anæmia. | 5 months. | 2 gr. 3 p. d. | Success. | Remedy given two weeks. |
| XI. | 32 | M. | Gen. atony fr. nurs'g. | 2 months. | 3 gr. 3 p. d. | Success. | " " |
| XII. | 21 | S. | Anæmia. | 6 months. | 1 gr. 3 p. d. | Failure. | Remedy given two weeks at two different periods. Suspicion of phthisis. Later, iron and cod liver oil, successful. |
| XIII. | 32 | M. | G. a. in obese person. | 2 months. | 2 gr. 3 p. d. | Success. | Remedy given every alternate week for six weeks. |
| XIV. | 25 | M. | Anæmia following nursing. | 3 months. | 2 gr. 3 p. d. Later, 4 gr. 3 p. d. | Success. | The large dose proved successful, after ten days' administration. |

*Note.*—In all but two of the above cases, the remedy occasioned more or less epigastric pain, substernal pressure and colicky pains.

*Conclusions.*—I. Permanganate of potash, in doses of from two to four grains, is an efficient emmenagogue, if administered for a period of not less than two weeks.

II. Its administration in doses large enough to be effectual is accompanied by severe pain, which frequently necessitates a discontinuance of the remedy, and hence impairs its value as an emmenagogue.

III. The most efficient method of administering the drug is in capsules, taken midway between meals, and followed by large draughts of some pure mineral water, like silurian.

2406 Prairie Ave.

